# Rhythmic Oscillations of Excitatory Bursting Hodkin-Huxley Neuronal Network with Synaptic Learning

**DOI:** 10.1155/2016/6023547

**Published:** 2016-03-17

**Authors:** Qi Shi, Fang Han, Zhijie Wang, Caiyun Li

**Affiliations:** Engineering Research Center of Digitized Textile & Apparel Technology, College of Information Science and Technology, Donghua University, Shanghai 201620, China

## Abstract

Rhythmic oscillations of neuronal network are actually kind of synchronous behaviors, which play an important role in neural systems. In this paper, the properties of excitement degree and oscillation frequency of excitatory bursting Hodkin-Huxley neuronal network which incorporates a synaptic learning rule are studied. The effects of coupling strength, synaptic learning rate, and other parameters of chemical synapses, such as synaptic delay and decay time constant, are explored, respectively. It is found that the increase of the coupling strength can weaken the extent of excitement, whereas increasing the synaptic learning rate makes the network more excited in a certain range; along with the increasing of the delay time and the decay time constant, the excitement degree increases at the beginning, then decreases, and keeps stable. It is also found that, along with the increase of the synaptic learning rate, the coupling strength, the delay time, and the decay time constant, the oscillation frequency of the network decreases monotonically.

## 1. Introduction

Neural systems could exhibit rhythmic oscillations, which are a type of synchronous state. Synchronization in neural systems is thought to be important for processing of sensory information and motor function [1], but the occurrence of synchronization in some specific areas of the brain may also be associated with some diseases, such as the epilepsy and Parkinson's disease [2]. Because of the importance of synchronization in neural systems, it has been studied for a long time from many aspects in neuroscience research [3–7].

There are up to 10^11^ neurons in human brain, and each neuron is connected to approximately 10^4^ other neurons. Neurons are coupled with each other by electrical or chemical synapses, and chemical synapses are dominant in quantity. The chemical synapse is related to the exchange of neurotransmitters between neurons and can be inhibitory or excitatory. Neurons coupled by different types of synapses constitute different networks, in which the dynamical behaviors can be very different [8]. Han et al. [9] found that the synchronization for inhibitory neuronal systems is more robust and stable than that for excitatory neuronal systems, so they investigated robust synchronization for a globally coupled inhibitory neuronal network. However, they did not study dynamical behaviors for excitatory neuronal systems, which should also be explored to reveal the underlying mechanisms of rhythmic oscillations in neural systems.

Synaptic plasticity is a prevalent feature of biological neural systems and considered to be critical for memory and learning functions of brains. Synaptic efficacy could be regulated by the plasticity at a variety of time scales, like from milliseconds to minutes. To study how synaptic plasticity works in neural systems, many synaptic learning rules, such as Hebbian learning rule and STDP rule, are proposed. And accordingly, the dynamics of neuronal systems under the influence of synaptic plasticity has been explored [10–13]. For example, Han et al. [13] investigated the dynamical properties of Newman-Watts (NW) small-world neuronal networks with a short-term synaptic plasticity named Oja rule and got some interesting findings for electrically and chemically coupled neuronal networks, respectively. But the study is insufficient, because synapses have more detailed structure which influences the dynamics of the neural systems a lot, especially for chemical ones.

This paper aims to find out how the synaptic learning and chemical synaptic parameters influence rhythmic oscillations in excitatory neuronal network. It is organized as follows. The model of a globally coupled excitatory bursting Hodkin-Huxley (HH) neuronal network is presented in Section 2. The results of simulations on the excitatory neuronal network, including the effects of the coupling strength and the synaptic learning rate and the effects of chemical synaptic parameters, are presented in Section 3. The conclusions are given in Section 4.

## 2. Model and Dynamics

The traditional Hodkin-Huxley (HH) model neuron only emits spikes. By incorporating a slow calcium ionic channel into the HH model, a modified model neuron which could emit bursts can be obtained [9]. In this paper, the modified HH model neuron is used to construct neuronal network.

### 2.1. Model Neuron

The equations and parameters of a single modified HH model neuron can be described as follows (the membrane potential is measured in mV and time in ms):

(1) CmdVdt=−gNam∞3hV−ENa−gKn4V−EK−gCaM2HV−ECa−gLV−EL+Iapp,

where the parameter *V* is the membrane potential of the modified model neuron. In (1), the activation or inactivation variables *h*, *n*, *M*, and *H* are associated with Na^+^, K^+^, and Ca^2+^ voltage-dependent ion currents, respectively, which changes quickly and satisfies the following steady-state functions:

(2a) dhdt=θαh1−h−βhh,

(2b) dndt=θαn1−n−βnn,

(2c) dMdt=τM−1−M+M∞,

(2d) dHdt=τH−1−H+H∞.

In (1), the activation variable *m*_*∞*_ is related to transient sodium current, which changes very fast and satisfies the steady-state function:

(3a) m∞=αmαm+βm,

(3b) αm=−0.1Vi+35exp⁡−0.1Vi+35−1,

(3c) βm=4exp⁡−Vi+6018.

In (2a)–(2d), the related variables satisfy the following functions:

(3d) αh=0.07exp⁡−Vi+5820,

(3e) βh=1exp−0.1Vi+28+1.

(3f) αn=−0.01Vi+34exp−0.1Vi+34−1,

(3g) βn=0.125∗exp⁡−Vi+4480.

(3h) M∞=11+exp⁡−Vi+57/6.2,

(3i) H∞=11+exp⁡Vi+81/4,

(3j) τM=0.612+1exp⁡−Vi+132/16.7+exp⁡Vi+16.8/18.2,

(3k) τH=exp⁡Vi+46766.6,Vi<−80 mV,28+exp⁡−Vi+2210.5,Vi≥−80 mV.

The values of parameters in (1), (2a), (2b), (2c), and (2d) are set as follows: *C*_*m*_ = 1 *μ*F/cm^2^, *g*_Na_ = 35 ms/cm^2^, *E*_Na_ = 55 mV, *g*_K_ = 9 ms/cm^2^, *E*_K_ = −90 mV, *g*_Ca_ = 3 ms/cm^2^, *E*_Ca_ = 120 mV, *g*_L_ = 0.1 ms/cm^2^, and *E*_L_ = −65 mV, *θ* = 5.

**Figure 1:**
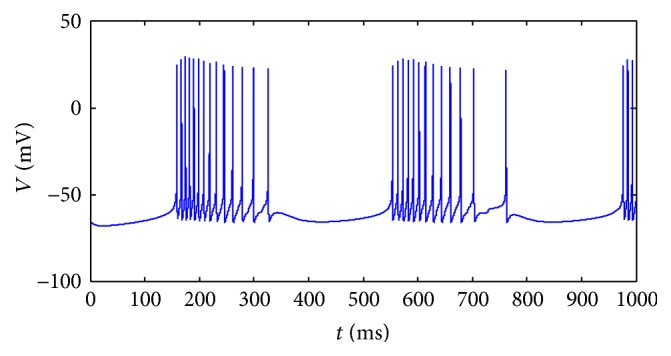
The firing pattern of a single neuron.

In (1), *I* ^app^ is injected current (in *μ*A/cm). Neurons could exhibit different firing patterns for different values of *I* ^app^. If *I* ^app^ belongs to [−0.95, −0.25], the modified HH neuron exhibits bursting behaviors (see Figure 1). The value of the injected current is set as −0.5 in this figure.

### 2.2. Neuronal Network Model

By using the above model neuron, we can set up a globally coupled neuronal network with 50 neurons. Considering that most of synapses are chemical ones and the weights of synapses are always changing, we apply a synaptic learning rule, Oja learning rule [13], to the chemical synapses in the neuronal network model. The globally coupled neuronal network is described as follows:

(4) CmdVidt=−gNam∞3hVi−ENa−gKn4Vi−EK−gCaM2HVi−ECa−gLVi−EL+Iiapp−σ∑j=150WijaijstVi−Esyn,

where the parameter *V*_*i*_ is the membrane potential of the *i*th neuron, *i* = 1,2,…, 50, *σ* is the maximal synaptic conductance, *a*_*ij*_ is the element of the adjacency matrix and equals 0 or 1 depending on whether there is a synapse between neurons *i* and *j*, and *E*_syn_ is the reversal potential.

In (4), *W*_*ij*_ is the value of the weight between neurons *i* and *j*, which changes according to the following equation:

(5) $$\begin{array}{c} \overset{•}{W_{ij}}=L*\arctan\left[\;V_{i}\left(\;V_{j}-V_{i}W_{ij}\;\right)\;\right], \end{array}$$

where *L* denotes the learning rate of the chemical synapse and *V*_*i*_ and *V*_*j*_ denote the membrane potentials of neuron *i* and neuron *j*, respectively.

In (4), *s*(*t*) is the level of openness of ion channels, *s*(*t*) = ∑_*m*_ *s*_*m*_(*t*), where *s*_*m*_(*t*) is associated with the *m*th spike of the presynaptic neuron, which is characterized by the following *α*-function:

(6) $$\begin{array}{c} s_{m}\left(\;t\;\right)=\left\{\begin{array}{cc} 0, & t<t_{m}^{f}+d\\ \frac{\left(exp\left(-\left(t-t_{m}^{f}-d\right)/\tau_{d}\right)-exp\left(-\left(t-t_{m}^{f}-d\right)/\tau_{r}\right)\right)}{\left(\tau_{d}-\tau_{r}\right)}, & t\geq t_{m}^{f}+d, \end{array}\right. \end{array}$$

where *t*_*m*_^*f*^ is the *m*th firing time of the presynaptic neuron and *τ*_*d*_, *τ*_*r*_, and *d* are the decay time constant, rise time constant, and synaptic delay of the synapse, respectively.

The chemical synapse can be excitatory or inhibitory. If *E*_syn_ is high enough, the synapse is excitatory; if *E*_syn_ is low enough, the synapse is inhibitory. In this paper, *E*_syn_ is set as 0 mV to make all the synapses excitatory.

## 3. Simulation Results

In this section, the dynamical properties of the excitatory neuronal network are studied. *I* ^app^ for each neuron is chosen randomly in [−0.5, −0.4] so that all the neurons can show different bursting behaviors. In the next simulations, the time step is 0.01 ms and the total simulation time is 1000 ms.

**Figure 2:**
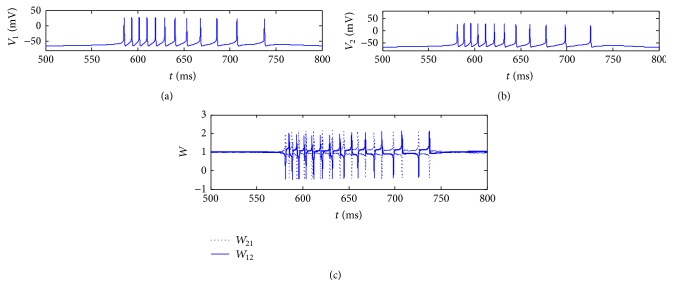
Spike trains of two arbitrarily chosen connected neurons in the network ((a), (b)) and synaptic weights between them in two directions, respectively (c).

We first explore the variations of synaptic weights under the influence of the synaptic leaning rule. Figures 2(a) and 2(b) show the spike trains of two arbitrarily chosen connected neurons in the network and Figure 2(c) shows their synaptic weights. The learning rate and the coupling strength are set as *L* = 2, *σ* = 0.05.

In order to see the variations in Figure 2 clearly, we extract some points in Figure 2 and list their values in Table 1. In Table 1, the values of the membrane potentials of the two neurons and the weights between them at 550 ms, 593.47 ms, 644.80 ms, 668.00 ms, 698.34 ms, and 750 ms are shown, respectively. It can be seen that, at 550 ms and 750 ms, both of the two neurons are at rest and the weights between them almost do not change; at 593.47 ms and 668.00 ms, neuron 1 is excited and neuron 2 is at rest, and obviously the weight from neuron 1 to neuron 2 (*W*_12_) increases and the weight from neuron 2 to neuron 1 (*W*_21_) decreases; at 644.80 ms and 698.34 ms, neuron 2 is excited and neuron 1 is at rest, and obviously the weight from neuron 2 to neuron 1 (*W*_21_) increases and the weight from neuron 1 to neuron 2 (*W*_12_) decreases.

So from Figure 2 and Table 1, we can come to the conclusion that the synaptic weights between two neurons do not change when both of them are in the state of rest, and if one neuron is excited and the other one is at rest, the weight from the excited neuron to the rest one is strengthened, and the weight from the rest one to the excited one is weakened. It means that, when ion currents are conducted between two neurons by the chemical synapses, the synaptic efficacy would be strengthened from the active neuron to the inactive one, and on the contrary, the synaptic efficacy would be weakened from the inactive one to the active one. This is plausible in real neural systems.

### 3.1. Effects of Coupling Strength and Synaptic Learning Rate

**Figure 3:**
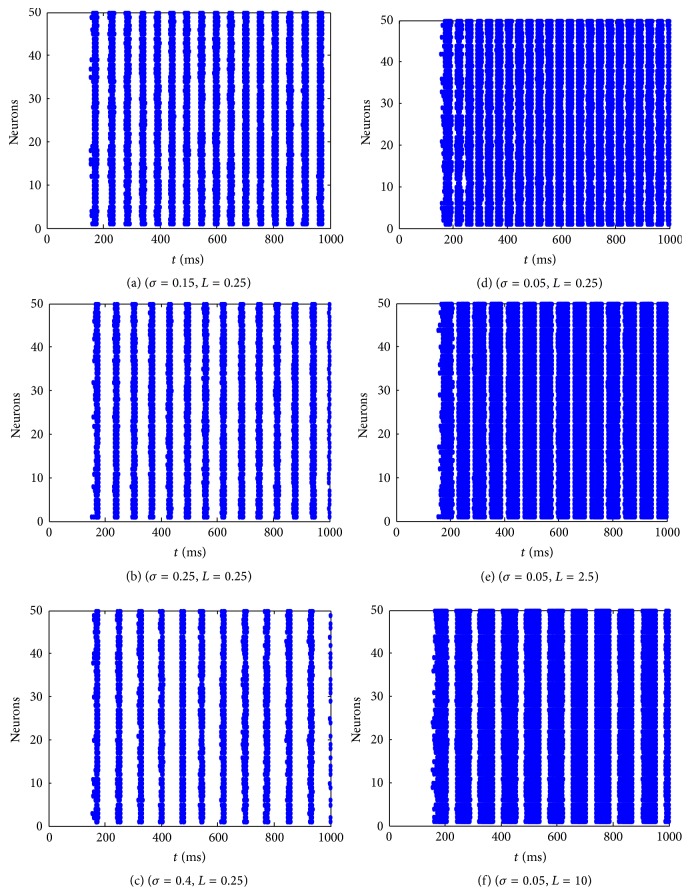
Some spatiotemporal patterns of the coupled excitatory neuronal network with different values of coupling strength and learning rate.

In order to see the dynamical properties of the whole network, some spatiotemporal patterns of the network with different values of coupling strength *σ* and learning rate *L* are plotted in Figure 3.

Figure 3 shows the effects of different parameters on the overall dynamics of the network, which can be divided into two groups. The first group of (a), (b), and (c) shows the effect of the coupling strength and it can be seen that the network with larger coupling strength is less excited. The second group of (d), (e), and (f) demonstrates the effect of the learning rate and it can be found that the neuronal network with *L* = 2.5 is much more excited than that with *L* = 0.25 and *L* = 10. However, it seems difficult to see the exact variation tendency of the influence of the parameters, so we need to use some measurements to carry out quantitative analysis.

In the next simulations, we use two measurements, excitement degree *D*_exc_ and oscillation frequency *F*_mean_, to describe the properties of the neuronal networks. All the results are obtained by average of 20-time repetition.

The measurement excitement degree is defined as *D*_exc_ = 〈*n*_exc_(*t*)〉, where *n*_exc_(*t*) indicates the proportion of the number of excited neurons in the neuronal network at the moment and 〈·〉 indicates averaging over time. This measurement reflects the extent of excitement of the neuronal network. Here, a neuron is thought to be excited once its spike exceeds a threshold, like −50 mV, or else it is thought to be inhibited. Larger value of the quantity *D*_exc_ means the neuronal network is more excited.

As the network activity is very rhythmic, we can also check the oscillation frequency of the neuronal network. The oscillation frequency *F*_mean_ can be defined as *F*_mean_ = (1/*N*)∑_*i*=1_^*N*^ *f*_*i*_, *f*_*i*_ = (*ϕ*_*i*_^*t*_2_^ − *ϕ*_*i*_^*t*_1_^)/2*π*(*t*_2_ − *t*_1_), where *f*_*i*_ is the bursting frequency of neuron *i* and *ϕ*_*i*_^*t*_2_^ and *ϕ*_*i*_^*t*_1_^ are the burst-phases at times *t*_2_ and *t*_1_ (*t*_2_ > *t*_1_), respectively.

By using the above two measurements, the dynamical properties of the excitatory neuronal network are studied. Firstly, we study the influence of the coupling strength and the learning rate on excitement degree and oscillation frequency. The parameters of delay time *d*, decay time *τ*_*d*_, and rise time *τ*_*r*_ are set as 8 ms, 12 ms, and 0.3 ms, respectively.

**Figure 4:**
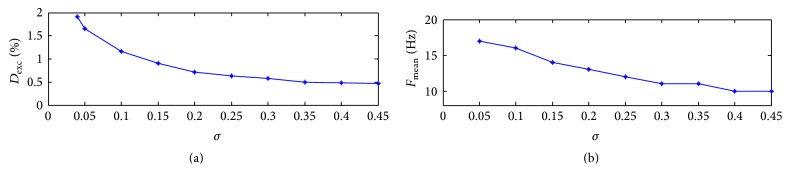
Effect of the coupling strength on excitement degree (a) and oscillation frequency (b) with *L* = 0.25.

Figures 4(a) and 4(b) display the effect of coupling strength on excitement degree and oscillation frequency, respectively. It can be seen that both excitement degree and oscillation frequency decrease monotonically along with the increase of the coupling strength. We may draw a conclusion that high coupling strength could decrease the excitement in a certain range.

**Figure 5:**
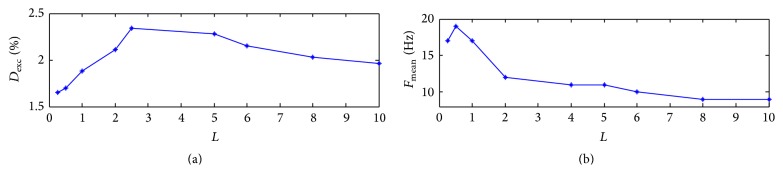
Effect of the learning rate on excitement degree (a) and oscillation frequency (b) with *σ* = 0.05.

Figures 5(a) and 5(b) show the effect of the learning rate on excitement degree and oscillation frequency for the network. It can be seen that excitement degree increases at first and then decreases along with the increasing learning rate. It achieves the maximum with *L* = 2.5. When it comes to the oscillation frequency (see Figure 5(b)), it increases at the very beginning and achieves the maximum 18 Hz and then decreases monotonously to 9 Hz at the end. It implies that the learning rate can regulate the excitement and oscillation periods of the network at a certain range.

### 3.2. Effects of the Chemical Synaptic Parameters

Then, we explore the influence of the synaptic parameters of chemical synapses. The values of the coupling strength, the learning rate, and the rise time are set as 0.25, 0.25, and 0.3 ms, respectively.

**Figure 6:**
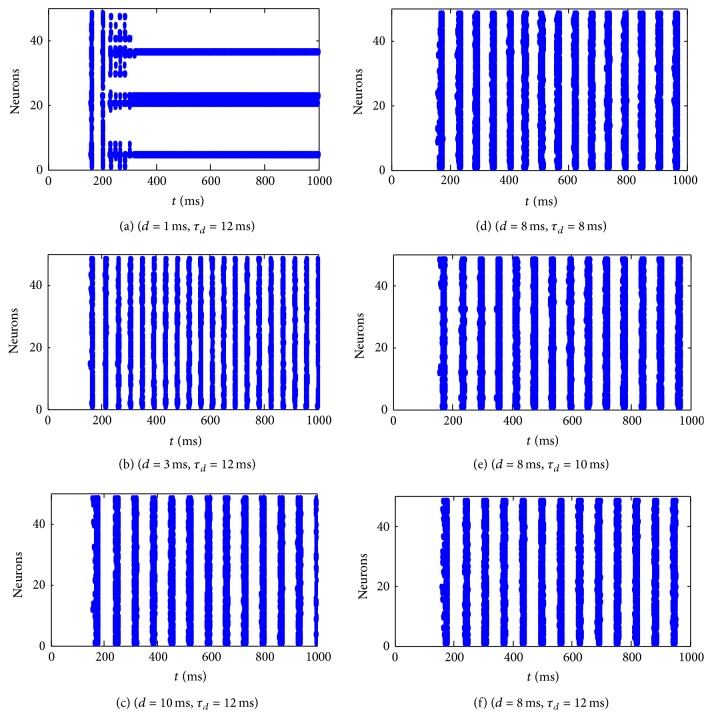
Some spatiotemporal patterns of the excitatory neuronal network under the different values of synaptic delay and decay time constant.

Figure 6 also can be divided into two groups. The first group of (a), (b), and (c) shows the effects of the delay on the overall dynamics of the network and it can be found that the network with delay time *d* = 3 ms is more active. The second group of (d), (e), and (f) demonstrates the effects of the decay time constant. Still, we need to quantify these phenomena.

**Figure 7:**
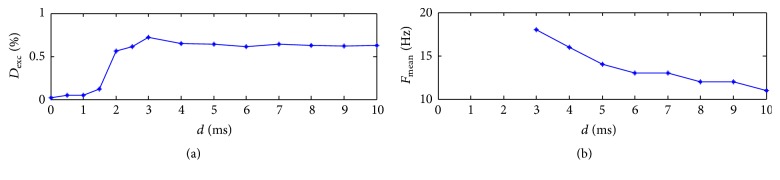
Effect of the synaptic delay on excitement degree (a) and oscillation frequency (b) with *τ*_*d*_ = 12 ms.

**Figure 8:**
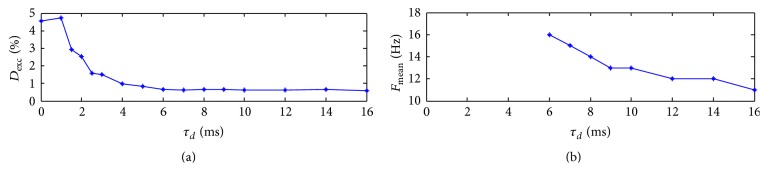
Effect of the decay time constant on excitement degree (a) and oscillation frequency (b) with *d* = 8 ms.

To study the effect of the delay time and the decay time constant, we plot Figures 7(a), 7(b), 8(a), and 8(b).

Figures 7(a) and 7(b) show the effect of the delay time on excitement degree and oscillation frequency for the network. The parameter of the decay time is set as *τ*_*d*_ = 12 ms. It can be seen from Figure 7(a) that excitement degree increases at first, then decreases a little, and then keeps stable along with the increasing synaptic delay. It achieves the maximum with the delay *d* = 3 ms. From Figure 7(b) it can be seen that the oscillation frequency decreases monotonically with the increasing synaptic delay. It implies that synaptic delay time can improve the excitement of the network but decrease periods of the rhythmic behaviors for neuronal networks.

The effects of the decay time constant on excitement degree and oscillation frequency for the network are shown in Figures 8(a) and 8(b). The value of the synaptic delay is set as *d* = 8 ms. It can be seen that the excitement degree decreases in general except for the very beginning and the oscillation frequency also decreases monotonically when the decay time constant is increasing. We may draw a conclusion that the decay time constant can weaken the excitement of the network and decrease periods of the rhythmic oscillations.

## 4. Conclusions and Discussions

The dynamics of an excitatory neuronal network with a synaptic learning rule are explored in this paper. It is found that both the coupling strength and the synaptic learning rate have great influence on the excitement degree and oscillation frequency of the neuronal network. The increasing of the coupling strength can weaken the extent of excitement, whereas increasing the synaptic learning rate makes the network more excited in a certain range. What is more, the increasing of both the coupling strength and the learning rate could decrease the oscillation frequency of the network. These results can be demonstrated well by the spatiotemporal patterns of the neuronal networks.

Besides, it is found that the synaptic parameters of delay time and decay time constant also have great influence on the excitement degree and oscillation frequency of the network. Along with the increasing of the delay time and the decay time constant, the oscillation frequency of the network decreases monotonically, and the excitement degree increases at the beginning, then decreases, and keeps stable.

In addition, we also check the situations for inhibitory neuronal networks. We may draw a conclusion that the coupling strength and the synaptic learning rate have little influence on the excitement of the network. Along with the increasing of the coupling strength and learning rate, the excitement degree and oscillation frequency change hardly. But large decay time and delay time can decrease the oscillation frequency of the network.

The results we obtained in this paper may be instructive for understanding the information processing mechanisms of neural systems and for the study of controlling synchronization in neural systems. It is known that human brain waves are sometimes rhythmic, which relate to human's certain behavior or brain diseases. So, the study of rhythmic oscillations can help us know about our brains and cure some diseases. By regulating some parameters of synapses, the rhythmic behaviors of neural systems could be altered.

## Figures and Tables

**Table 1:** The values of some extracted points as examples in Figure 2.

Time (ms)	550.00	593.47	644.80	668.00	698.34	750
*V*_1_ (mV)	−62.6378	28.6678	−62.4997	24.6842	−59.6050	−60.6416
*V*_2_ (mV)	−62.8390	−55.9874	26.0039	−60.5042	22.8384	−58.9853
*W*_12_	1.0189	1.3345	−0.4245	1.4635	−0.2923	0.9867
*W*_21_	0.9811	−0.3117	1.4746	−0.3372	1.5400	1.0451
